# Supplementary material for: Genetic comparisons of interleukin-17 reveal a framework for complex signaling evolution
Source: bioRxiv. 2026 Apr 14:2026.04.13.718218. Preprint. [Version 1] doi: 10.64898/2026.04.13.718218 (PMC13105070; doi:10.64898/2026.04.13.718218)
Supplement: Supplement 3 [file media-3.zip › Supplemental files for paper/ERC/IL17xIL17_comparisons/IL17B_IL17RB.pdf]

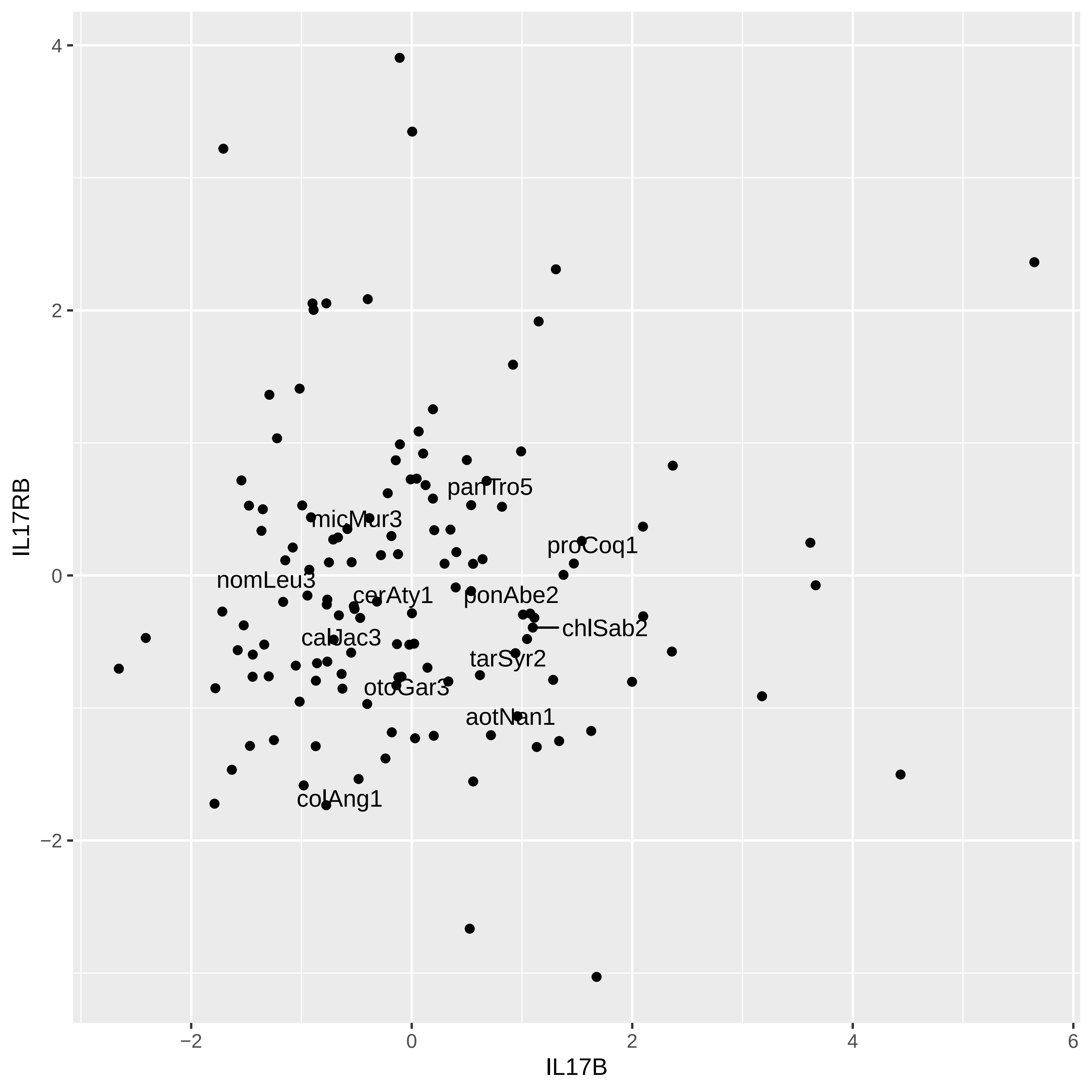

panTro5
micMur3
proCoq1
nomLeu3
cerAty1
ponAbe2
chlSab2
calJac3
tarSyr2
otoGar3
aotNan1
colAng1
4
2
0
−2
−2
0
2
4
6
IL17RB
IL17B
